# Patient-Specific CT-Based Fluid-Structure-Interaction Aorta Model to Quantify Mechanical Conditions for the Investigation of Ascending Aortic Dilation in TOF Patients

**DOI:** 10.1155/2020/4568509

**Published:** 2020-08-08

**Authors:** Heng Zuo, Yunfei Ling, Peng Li, Qi An, Xiaobo Zhou

**Affiliations:** ^1^Biomedical Big Data Center, West China Hospital, Sichuan University, Chengdu 610041, China; ^2^School of Mathematics, Sichuan Normal University, Chengdu 610068, China; ^3^Department of Cardiovascular Surgery, West China Hospital, Sichuan University, Chengdu 610041, China; ^4^Center for Computational Systems Medicine and School of Biomedical Informatics, University of Texas Health Science Center at Houston, Houston, TX, USA

## Abstract

**Background:**

Some adult patients with Tetralogy of Fallot (TOF) were found to simultaneously develop ascending aortic dilation. Severe aortic dilation would lead to several aortic diseases, including aortic aneurysm and dissection, which seriously affect patients' living quality and even cause patients' death. Current practice guidelines of aortic-dilation-related diseases mainly focus on aortic diameter, which has been found not always a good indicator. Therefore, it may be clinically useful to identify some other factors that can potentially better predict aortic response to dilation.

**Methods:**

20 TOF patients scheduled for TOF repair surgery were recruited in this study and were divided into dilated and nondilated groups according to the *Z* scores of ascending aorta diameters. Patient-specific aortic CT images, pressure, and flow rates were used in the construction of computational biomechanical models.

**Results:**

Simulation results demonstrated a good coincidence between numerical mean flow rate at inlet and the one obtained from color Doppler ultrasonography, which implied that computational models were able to simulate the movement of the aorta and blood inside accurately. Our results indicated that aortic stress can effectively differentiate patients of the dilated group from the ones of the nondilated group. Mean ascending aortic stress-P1 (maximal principal stress) from the dilated group was 54% higher than that from the nondilated group (97.97 kPa vs. 63.47 kPa, *p* value = 0.044) under systolic pressure. Velocity magnitude in the aorta and aortic wall displacement of the dilated group were also greater than those of the nondilated group with *p* value < 0.1.

**Conclusion:**

Computational modeling and ascending aortic biomechanical factors may be used as a potential tool to identify and analyze aortic response to dilation. Large-scale clinical studies are needed to validate these preliminary findings.

## 1. Introduction

Tetralogy of Fallot (TOF) is a congenital heart defect which involves four anatomical abnormalities of the heart: ventricular septal defect, pulmonary infundibular stenosis, overriding aorta, and right ventricular hypertrophy. In the United States, the prevalence of TOF is 3.9 per 10,000 live births and accounts for 7-10% of congenital heart diseases [1]. Some adult patients with congenital heart disease were found to simultaneously develop ascending aortic dilation [2–4]. A dilatation of all wall layers of the aorta of more than 50% in comparison to the normal diameter is defined as the ascending aortic aneurysm [5], which is generally indolent and asymptomatic until presentation with catastrophic complications of rupture and dissection. When a rupture or dissection occurs, it is fatal in a large proportion of patients. During the acute phase of aortic dissection, more than 50% of patients die if not treated surgically, and the mortality of those treated ranges from 5% to 30% despite the significant improved operative strategies [6–8].

The risk of aortic dissection is known to highly correlate with increasing aortic diameter. Current practice guidelines have recommended elective aortic repair at a diameter of 5.5 cm because the risk of rupture of an aneurysm is found to increase dramatically with ascending aortic diameter greater than 6 cm [9–13]. However, recent studies have found that many patients with acute ascending aortic dissection have aortic diameters of <5.5 cm at hospital presentation [14, 15]. Moreover, Ming et al. conducted a systematic review and meta-analysis about the natural history of ascending aortic aneurysm and found the conclusions about the conventional risk factors, such as aortic diameters, not only varied significantly but were often contradictory among studies [16]. For example, 4 studies [17–20] identified that aortic dilation had no association with baseline aortic diameter, while 2 studies [21, 22] reported higher annual aortic growth rate with lower baseline aortic diameter. Therefore, more and more researches focus on searching for more reliable risk factors of ascending aortic dilation rather than aortic diameter only. Geisbusch et al. proposed computed tomography volume measurements which provide an objective method for ascertaining aortic size and monitoring expansion [23]. Donato Aquaro et al. used the maximum rate of systolic distension (MRSD) as an index of aortic wall properties and found MRSD was a valuable predictor for progression in ascending aortic dilation [24]. Alreshidan et al. used speckle tracking echocardiography to estimate aortic stiffness in vivo and proved that the stiffness was helpful to determinate the risk of complications in patients with aortic diseases [25]. Pasta et al. used CT angiographic imaging to estimate patient-specific in vivo strain fields and found stiff behavior for the aneurysmal aorta compared with that of the healthy ascending aorta [26]. Farzaneh et al. proposed a noninvasive method by using gated CT scans to identify the patient-specific local extensional stiffness of aortic walls, which was found to be highly correlated with the rupture risk criterion [27, 28].

With the rapid improvement of computer and medical imaging technologies, image-based computational models have been widely used in the research of cardiovascular diseases. Youssefi et al. used computational fluid-only biomechanical models to investigate the impact of inflow velocity profiles on hemodynamics of the thoracic aorta [29]. Condemi et al. performed patient-specific computational fluid dynamics analysis to obtain the effect of hemodynamics on the ascending thoracic aortic aneurysm risk of rupture [30] and also investigated the hemodynamic alterations for a patient who was treated by only ascending aorta replacement preserving the BAV [31]. Underhill et al. constructed image-based computational models by integrating plaque morphology, components, and mechanical stress/strain conditions to assess the vulnerability of human atherosclerotic carotid plaques [32]. According to some studies associated with aortic diseases, biomechanics of aortic tissue has been found to be a possible good predictor of the histologic integrity of the aortic wall [17, 33–35]; therefore, biomechanics might be a better risk factor of aortic diseases than aortic diameter. The objective of this study is to construct a computational biomechanical model by combining patient-specific CT scans, aortic pressure measurements, color Doppler ultrasonography, and aortic tissue properties for a better understanding of mechanical environment of the ascending aorta, which may provide a more useful insight about the ascending aortic dilation.

## 2. Data Acquisition, Models, and Methods

### 2.1. Data Acquisition

Twenty TOF patients scheduled for TOF repair surgery were recruited in this study, which was approved by the review board on human subject research (West China Hospital, West China School of Medicine), and informed consent was obtained.

**Figure 1:**
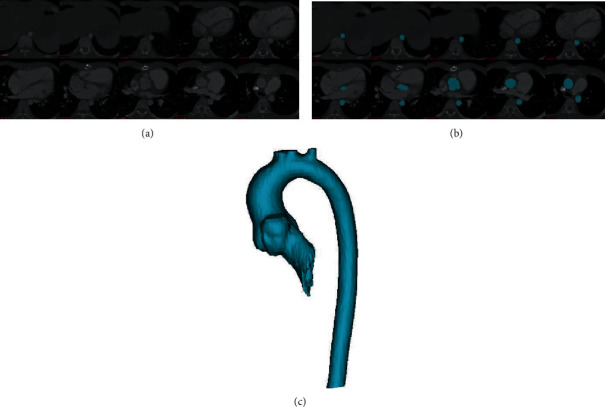
CT-based model construction process. (a) Selected CT slices from a patient, under diastolic pressure; (b) segmented results of the aorta; (c) reconstructed 3D geometry of the aorta.

ECG-gated cardiac computed tomography (CT) scans were performed in one cardiac cycle for each patient, and scans of approximately 10 time points were obtained. The CT scans of each time point contained around a total of 70 slices (slice thickness was 3 mm) covering the whole cardiac structure and ascending aorta, and scans under diastolic and systolic pressure were used in the model construction. 3D geometry of the aortic root and ascending aorta was obtained from CT scans using thresholding-segmentation technique implemented in the Mimics (Materialise, Belgium). The lumen of the aortic root and ascending aorta were acquired by using Mimics segmentation module “CT Heart Segmentation” with the recommended parameters. Due to the image qualities, patient-specific aortic wall is hard to obtain and a uniform wall thickness 2.0 mm was used for all the patients in this paper.  Figure 1 shows selected CT scans, segmented results of the aorta, and the corresponding 3D reconstructed geometry for one patient in our dataset. The fluid-structure-interaction (FSI) finite element (FE) model of the ascending aorta would be constructed based on the CT-based 3D geometry and color Doppler ultrasonographic flow information to quantify the mechanical properties of aorta.

Patients' ascending aortic diameters were calculated from the 3D reconstructed geometries and converted to their corresponding *Z* scores by using a web-based calculation tool (http://zscore.chboston.org) which had collected baseline data over the past 12 years [36]. On account of the inclusion of patient-specific gender, age, and body surface area (BSA), *Z* score has the advantage in determining whether an aorta is within normal size limits. Rather than raw aortic diameter, aortic *Z* score is more accurate in determining whether true pathology exists. We evenly divided all the patients into 2 groups according to their *Z* scores of ascending aortic diameters, where the group with larger *Z* scores was defined as the dilated group and the one with smaller *Z* scores was the nondilated group. The patient-specific systolic and diastolic blood pressure was measured at the same time when performing CT scanning, and they were used as boundary conditions in the simulations. Patient-specific color Doppler ultrasonographic imaging of blood flow in the ascending aorta was obtained and used in the determination of patient-specific boundary condition.

Patients' demographic information, aorta diameters and corresponding *Z* scores, systolic and diastolic blood pressures, and blood properties are summarized in Table 1. Continuous variables (such as age, aorta diameter, blood viscosity, and pressure) were summarized as mean ± SD and compared between the outcome groups using an unpaired Student *t*-test. Categorical variables (such as gender) were compared between different groups using a nonparametric test.

### 2.2. The Anisotropic Ascending Aorta Model

The aorta material was assumed to be hyperelastic, anisotropic, nearly incompressible, and homogeneous. The governing equations for the structure model are as follows:

(1) $$\begin{array}{c} \rho \frac{\partial^{2}u_{i}}{\partial t^{2}}=\frac{\partial \sigma_{ij}}{\partial x_{j}},\;i=1,2,3,\\ \varepsilon_{ij}=\frac{1}{2}\left(\frac{\partial u_{j}}{\partial a_{i}}+\frac{\partial u_{i}}{\partial a_{j}}\right)+\underset{l}{\sum }\frac{\partial u_{l}}{\partial a_{i}}\frac{\partial u_{l}}{\partial a_{j}},\;i,j=1,2,3. \end{array}$$

Here, *σ* is the stress tensor, *ε* is Green's strain tensor, *u* is the displacement, and *ρ* is the material density.

The nonlinear Mooney-Rivlin model was used to describe the aorta properties, which has been widely used to model anisotropic hyperelastic organs including ventricular tissue [37] and vessel [38]. The strain energy function for the anisotropic modified Mooney-Rivlin model is as follows [39]:

(2) $$\begin{array}{c} W=c_{1}\left(I_{1}-3\right)+c_{2}\left(I_{2}-3\right)+D_{1}\left[\exp\left(D_{2}\left(I_{1}-3\right)\right)-1\right]+K_{1}/\left(K_{2}\right)\exp\left[K_{2}{\left(I_{4}-1\right)}^{2}-1\right],\\ I_{1}=\sum C_{ii},\\ I_{2}=\frac{1}{2}\left[I_{i}^{2}-C_{ij}C_{ij}\right], \end{array}$$

where *I*_1_ and *I*_2_ are the first and second strain invariants, *C* = [*C*_*ij*_] = *X*^*T*^*X* is the right Cauchy–Green deformation tensor, *X* = [*X*_*ij*_] = [*∂x*_*i*_/*∂a*_*j*_] (*x*_*i*_ is the current position, *a*_*i*_ is the original position), *I*_4_ = *C*_*ij*_(**n**_f_)_*i*_(**n**_f_)_*j*_, **n**_f_ is the fiber direction which was set to circumferential in our study. *c*_*i*_, *K*_*i*_, and *D*_*i*_ are material parameters. In our models, initial values of material parameters were obtained by fitting the experimental stress-stretch data of human aorta samples [40] with the goodness of fit (*R*^2^) 0.73: *c*_1_ = −525.16 kPa, *c*_2_ = 165.9 kPa, *D*_1_ = 231.8 kPa, *D*_2_ = 3.5, *K*_1_ = 33.4 kPa, and *K*_2_ = 12.6. The patient-specific structure-only ascending aorta model was constructed using the geometry under diastolic pressure; then, the pressure difference between systolic and diastolic pressures was applied as boundary condition on the inner surface of the aorta to pressurize the aorta to its shape under systolic pressure. Patient-specific material parameters were acquired through adjusting initial material parameters by one ratio to match the numerical volume of the ascending aorta under systolic pressure with the one calculated from CT scans (relative error < 5%). The normal stress on the outer aorta surface was assumed to be zero. On the inner aorta surfaces, we applied fluid-structure-interaction boundary conditions.

### 2.3. Blood Model

**Figure 2:**
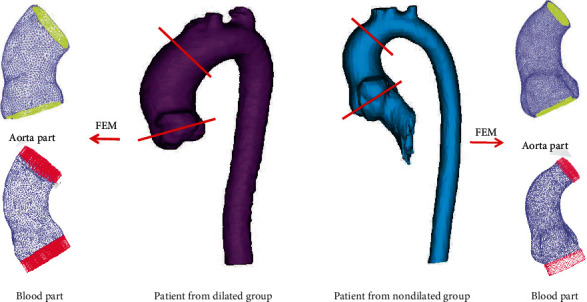
In the middle: 3D reconstructed aorta of two representatives from each group. On the left: structure mesh (up) and fluid mesh with imposed pressure conditions (down) of the dilated group representative. On the right: structure mesh (up) and fluid mesh with imposed pressure conditions (down) of the nondilated group representative.

**Figure 3:**
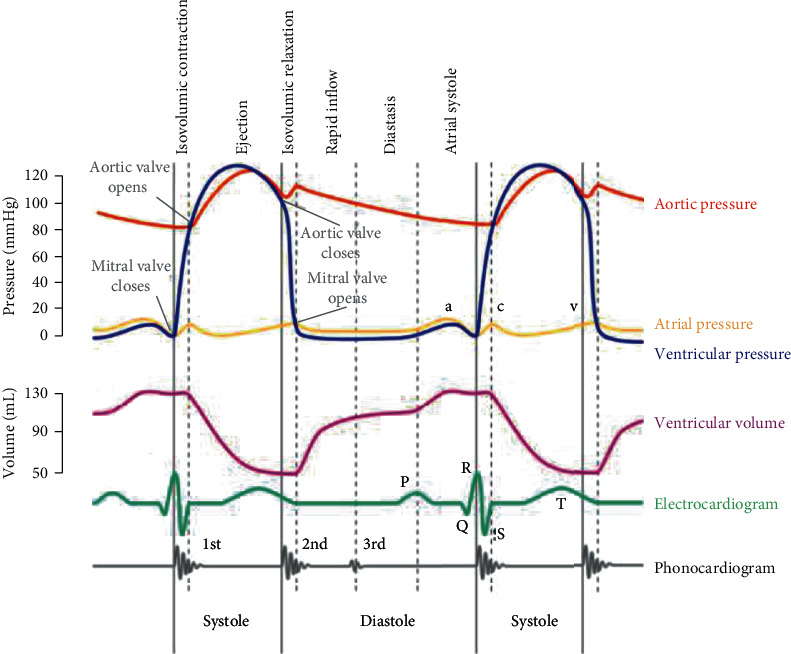
Prescribed pressure curve. Aortic pressure curve (the red curve) in the Wiggers diagram was adjusted according to patient-specific diastolic and systolic pressures and used as the pressure curve in the simulations.

Blood flow was assumed to be laminar, Newtonian, and incompressible. The Navier-Stokes equations with arbitrary Lagrangian-Eulerian formulation were used as the governing equations. No-slip conditions and natural traction equilibrium conditions were assumed at fluid-structure interfaces, pressure curves were adjusted according to patient-specific blood pressure, and flow rate information obtained from color Doppler ultrasonography was imposed at inlet (location of the aortic valve) and outlet (intersection between the ascending aorta and the aortic arch) of the aorta (see Figure 2).

(3) $$\begin{array}{c} \rho \left(\frac{\partial u}{\partial t}+\left(\left(u–ug\right)\cdot \nabla \right)u\right)=-\nabla p+\mu \nabla^{2}\;u,\\ \nabla \cdot u=0,\\ {\left.u\right|}_{\Gamma }=\frac{\partial x}{\partial t},\\ p\left|{}_{\text{inlet}}\right.=p_{\text{in}}\left(t\right),\\ p\left|{}_{\text{outlet}}\right.=p_{\text{out}}\left(t\right),\\ \sigma_{ij}^{\text{f}}\cdot n_{j}\left|{}_{\text{interface}}\right.=\sigma_{ij}^{\text{s}}\cdot n_{j}\left|{}_{\text{interface}}\right., \end{array}$$

where **u** and *p* are the fluid velocity and pressure, **u**_*g*_ is the mesh velocity, *μ* is the dynamic viscosity, *ρ* is the density (set to 1 g·cm^−3^), Γ stands for the aorta inner boundary, *σ* is the stress tensor (superscript letters “f” and “s” indicate fluid and solid materials, respectively), *ε* is the strain tensor, and **v** is the solid displacement vector. Aortic pressure curve in the Wiggers diagram (Figure 3) was adjusted according to patient-specific diastolic/systolic blood pressure and used as *p*_in_(*t*) prescribed at the inlet of the fluid domain, and the pressure condition at the outlet *p*_out_(*t*) was obtained by adjusting *p*_in_(*t*) to match the numerical mean velocity magnitude at inlet with the one measured by color Doppler ultrasonography (relative error < 5%). Patient-specific blood viscosity *μ* was calculated from the following the equation [41]:

(4) $$\begin{array}{c} \mu \;\left(\text{cPoise}\right)=0.12\text{ hematocrit }\left(\%\right)+0.17\text{ total plasma protein }\left(\text{g}/\text{L}\right)-2.07. \end{array}$$

### 2.4. Preshrink Process

Numerical simulation needs to start from an initial condition where the initial aorta shape, blood pressure, and stress/strain distributions were provided. Unfortunately, it is hard to measure stress distributions of soft tissues in vivo; therefore, our numerical simulations would start from zero-load state when blood pressure in the aorta was zero so that initial stress and strain distributions in the aorta were approximately considered as zero. However, CT scans were performed under the in vivo condition where the aorta was pressurized; thus, the zero-load shape was not obtained directly from CT scans. In our model construction process, a preshrink process was applied to the in vivo aorta shape to generate the zero-load shape. The initial shrinkage rate for the aorta was set to 5%, and diastolic blood pressure was applied so that the zero-load aorta shape would regain its in vivo morphology. The shrinkage rate was adjusted until pressurized numerical aorta shape highly agreed with CT scans (the relative error between the numerical volume of the ascending aorta section and the one calculated from CT scans < 5%). Without this preshrink process, the actual computing domain would be greater than the actual aorta due to the initial expansion when pressure was applied.

### 2.5. Geometry-Fitting Mesh Generation and Solution Method

Due to the complex irregular geometries of the ascending aorta, a geometry-fitting mesh generation technique [42] was adopted to generate meshes for our models. The fully coupled FSI model was solved by ADINA (ADINA R&D, Watertown, MA) using unstructured finite elements and the Newton–Raphson iteration method. Mesh analysis was performed by decreasing mesh size by 10% (in each dimension) until solution differences in maximal stress-P1/strain-P1 predictions were less than 2%, where stress-P1 and strain-P1 mean the maximal principal stress and strain, respectively. In our models, the optimal element size in each dimension was around 0.5 mm; the final number of tetrahedral meshes was about 3000 for the fluid domain and about 2000 for the solid domain.

### 2.6. Statistical Analysis

Due to the small sample size, the Shapiro-Wilk test was used to test if the data satisfied the normal distribution. Also, Levene's test was used to determine if variances for a variable calculated from two different groups were equal. Based on the results of Shapiro-Wilk and Levene's tests, the appropriate test for the comparison of means, such as Student's *t*-test or unequal variances *t*-test, was chosen to compare the differences in the numerical mechanical results between the nondilated and dilated groups.

## 3. Results

### 3.1. Blood Flow Dynamics in the Ascending Aorta

**Figure 4:**
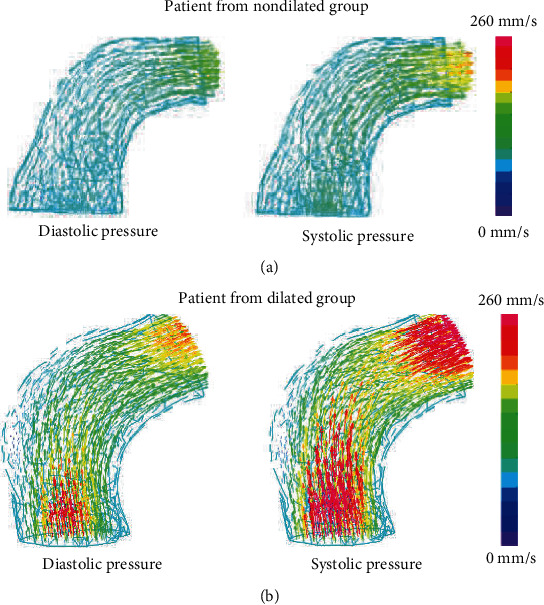
Flow distributions of the representative models: (a) flow distributions of a patient from the nondilated group at diastolic pressure (left) and systolic pressure (right); (b) flow distributions of a patient from the dilated group.

One patient was chosen from each group to represent flow distributions in the aorta; the streamlines of blood velocity fields in the two patients under systolic/diastolic pressure are shown in Figure 4. Numerical mean and inlet velocity magnitude in the ascending aorta of all the patients were summarized in Table 2, where inlet velocity means the average velocity magnitude over the inlet surface. It is worth mentioning that numerical volumes of the ascending aortic segment were compared with the one measured by CT scans, and relative errors of volume for all patients were less than 8%. The results of Shapiro-Wilk and Levene's test showed that both mean and inlet velocity magnitudes in each group satisfied the normal distribution and no significant different variances were found between the groups. Therefore, Student's *t*-test was appropriate to compare the means of velocity magnitudes between two groups. The results indicated that the dilated group had larger velocity magnitudes than the nondilated group, but the differences were not significant due to 5% level of significance. Inlet velocity magnitudes in the dilated group were greater than those in the nondilated group at the time of both systolic and diastolic pressure (*p* value: 0.058 for systolic pressure time and 0.097 for diastolic pressure time).

### 3.2. Mechanical Analysis of the Ascending Aorta

#### 3.2.1. Displacement Analysis

**Figure 5:**
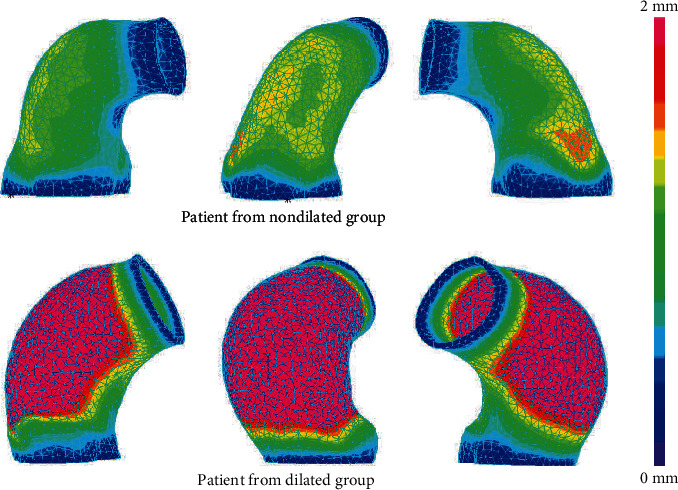
Displacement distributions of the representative models: the top row shows the displacement distributions of a patient from the nondilated group in three different views; the bottom row shows the displacement distributions of a patient from the dilated group. The same color band used to better present the differences between the two models.

Representative models' displacement distribution at the time of systolic pressure was presented in Figure 5. All patients' mean and maximal displacement values and corresponding test results were summarized in Table 3. Student's *t*-test results showed that no significant differences were found in mean displacement at the time of systolic pressure between the two groups (*p* value: 0.056). However, maximal systolic pressure displacement of the dilated group was found to be significantly larger than that of the nondilated group through Student's *t*-test with *p* value 0.038.

#### 3.2.2. Internal Stress Analysis in the Ascending Aorta

**Figure 6:**
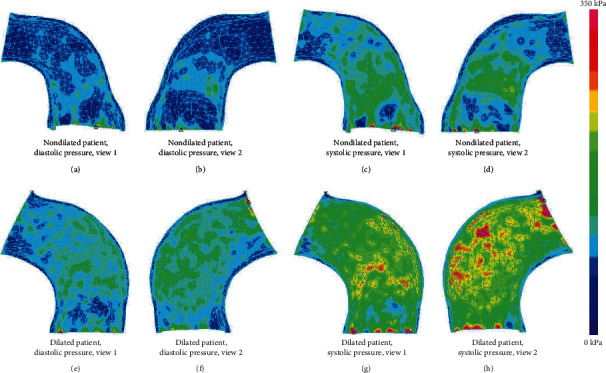
Stress distributions of the representative models: (a, b) the stress distributions of a patient from the nondilated group at the time of diastolic pressure, (c, d) the stress distributions of a patient from the nondilated group at the time of systolic pressure, (e, f) the stress distributions of a patient from the dilated group at the time of diastolic pressure, and (g, h) the stress distributions of a patient from the dilated group at the time of systolic pressure. The same color band used to better present the differences between the two models.

Figure 6 shows stress-P1 distributions of representative models for two different groups, and stress-P1 means the maximal principal stress. Average stress-P1 values in the ascending aorta of the 20 patients were summarized in Table 4. Mean stress-P1 values of the dilated group were significantly larger than those of the nondilated group at the time of systolic pressure with *p* value 0.044. Diastolic pressure mean stress-P1 of the dilated group was highly greater than that of the nondilated group with *p* value 0.089.

## 4. Discussion

In previous researches about TOF patients, while main attentions were paid on RV pathology including RV hypertrophy, pulmonary regurgitation, and RV outflow tract obstruction, few attentions were paid on LV. However, more and more TOF patients were found to have ascending aortic dilation of different levels in recent clinical observations. Severe aortic dilation would lead to some aortic diseases, such as aortic aneurysm and dissection, which seriously affect patients' living quality and even cause patients' death. Conventional clinical guidelines of aortic dilation only rely on the aortic diameter and ignore the tissue properties of the aorta which have been proved to own better abilities in prediction of aortic dilation progression especially in patients with small-to-moderate size ascending aortas [24]. Considering the good performances of computational biomechanical modeling in the investigations of cardiovascular diseases, we proposed image-based computational models in this study to simulate the movement of the ascending aorta and blood inside to obtain a better understanding of ascending aortic mechanics. To our knowledge, this is the first study applying biomechanical computational modeling in the research of ascending aortic dilation for TOF patients.

This study provides proof of concept that computational modeling based on patient-specific CT images can be used to simulate ascending aorta response to aortic dilation. Computational biomechanical models integrate patient-specific CT images, aortic pressure measurements, tissue properties, and color Doppler images through well-established equations of motion, constitutive equations of materials, and finite element method. Simulation results were patient-specific by comparing the numerical results with the ones obtained from clinical measurements. These complex computational models have provided new insights into mechanical environment (stress, strain, and flow distributions) in the ascending aorta, which is able to provide more information than aortic diameter only. In our simulations, sometimes patients with larger ascending aortic diameters were found to present lower stress in the aorta. For instance, patient #2 (Table 1) with ascending aortic diameter 27.18 mm had mean stress-P1 of the whole ascending aorta 89.44 kPa at systolic pressure and 55.33 kPa at diastolic pressure, while patient #8 with ascending aortic diameter 30.32 mm had mean stress-P1 71.67 kPa at systolic pressure and 41.77 kPa at diastolic pressure. The phenomenon happening indicated that diameter-only criterion for aortic dilation was insufficient. In our study, we divided the patients into the dilated and nondilated groups according to *Z* scores rather than raw measurements of ascending aorta diameters, since *Z* scores have been proven more capable to reflect the dilation degree of the aorta than raw aorta diameter measurements. Differences in *Z* scores between the dilated and nondilated groups were highly significant (*p* value < 0.01), while raw diameter measurements were not significantly different between the two groups (*p* value 0.051). According to numerical results, the dilated group was more likely to expand than the nondilated group. Meanwhile, the dilated group also tended to have larger stress than the other group indicating larger probability of vessel rupture for the dilated group. The mean flow rates in the ascending aorta of the dilated group were found to be larger than the ones of the nondilated group. Larger flow rates indicated the larger acting force of blood on the vessels which may cause the aortic dilation. In a word, the biomechanical model is a complex combination of aortic geometry, tissue property, and pressure which could provide more intrinsic insights into aortic dilation, dissection, and rupture. Moreover, the biomechanical models are able to display the distribution of mechanical parameters (see Figures 4–6) which is helpful in determination of focal area due to dilation.

The current study adds computational modeling as a new investigative tool and stress as new potential predictors for ascending aortic dilation. To obtain the mechanics of aortas as correctly as possible, the model assumptions were set to as close to reality as possible and the data including aortic geometry, pressure, and flow rate were all patient-specific. The results of this computational analysis study were intriguing. In particular, stress shows the most significant differences between the aortic dilated and nondilated groups, which also implies that aortic stress may be a good predictor of aorta response to aortic dilation. From a pathophysiologic perspective, these findings are plausible given that stress more accurately reflects the functional status of the aortas as compared with aortic diameter. From a clinical perspective, most published criteria for ascending aortic dilation have focused on aortic diameter. However, the results of this study suggest that aortic stress is also and may be more helpful in identifying the aortic tissue status, therefore informing the decision of clinical intervention than relying on aortic diameter alone.

This study is a preliminary work applying biomechanical modeling in the investigations of ascending aortic dilation. Several improvements can enhance our work in the future for better accuracy and applicability: (1) sample size needs to be enlarged to validate the findings in this study; (2) the addition of aortic valve mechanics to the model may improve accuracy with regard to timing of valve opening and closure and allow incorporation of aortic valve regurgitation; (3) in vivo measurements of tissue properties will be very desirable for improved accuracy of our models; (4) the uniqueness of tissue parameters is difficult to be guaranteed given the limited experimental data; (5) residual stress/strain which is defined as the initial stress/strain in the solid under zero-load state should be considered as nonzero for more accurate simulation results; (6) the addition of patient-specific aortic wall thickness will improve the accuracy of our simulations.

## 5. Conclusion

In this study, patient-specific biomechanical computational models of 20 TOF patients undergoing ascending aortic dilation of different levels were constructed by comparing numerical volume of the ascending aortic segment with the ones obtained from CT scans. Simulation results demonstrated that aortic stress, flow rates, and displacement were able to differentiate the patients with dilated ascending aorta from the nondilated ones, which implied that these factors may be highly correlated with aortic dilation and even the cause of dilation. These findings provide a basis for future studies aimed at validating these results in larger groups of patients and further refinements of the computational modeling to improve its accuracy.

## Figures and Tables

**Table 1:** Patients' demographic information, ascending aorta diameters, blood pressures, and blood properties.

	Age (year)	Sex	Height	Weight	SBP (mmHg)	DBP (mmHg)	AAD (mm)	AAD *Z*-score	Viscosity (cPoise)
Nondilated group									
P1	6.9	F	118	19.0	108	62	17.59	0.64	4.28
P2	21.8	M	163	50.0	130	85	27.18	1.73	10.01
P3	8.7	M	126	22.0	107	61	20.69	1.76	4.08
P4	8.4	M	119	20.0	98	64	20.16	1.86	6.73
P5	6.1	M	110	18.0	105	59	20.12	2.34	6.48
P6	21.3	F	150	45.0	105	56	30.32	3.13	6.07
P7	15.2	M	162	40.0	112	62	25.29	3.92	6.08
P8	15.1	M	170	52.0	95	58	30.32	4.11	4.14
P9	20.3	M	171	65.0	140	96	35.43	4.12	7.18
P10	12.0	M	138	31.5	88	57	29.76	4.15	2.78
Mean ± SD	13.5 ± 6.1		143 ± 23	36.3 ± 16.6	109 ± 16	66 ± 13	25.68 ± 5.86	2.78 ± 1.27	5.78 ± 2.06
Dilated group									
P11	7.6	F	129	20.0	79	50	20.60	5.81	3.82
P12	7.8	M	113	16.0	109	74	25.70	5.88	7.22
P13	10.4	F	125	23.0	105	60	30.89	7.03	7.27
P14	8.8	M	115	20.0	105	70	27.79	7.35	8.83
P15	19.9	M	166	46.0	133	77	40.17	7.45	8.59
P16	13.0	M	157	37.0	118	54	35.96	7.82	3.69
P17	13.2	M	152	28.0	129	69	37.07	8.66	8.30
P18	19.3	M	170	45.0	99	63	39.26	9.72	7.64
P19	8.6	F	120	15.0	90	60	28.71	11.36	4.74
P20	6.9	F	106	15.0	99	40	29.17	11.90	4.10
Mean ± SD	11.6 ± 4.8		135 ± 24	26.5 ± 12.0	107 ± 17	62 ± 11	31.53 ± 6.38	8.30 ± 1.27	6.42 ± 2.09
*p* value	0.42		0.49	0.15	0.76	0.45	0.051	<0.01	0.50

SBP: systolic blood pressure; DBP: diastolic blood pressure; AAD: ascending aortic diameter.

**Table 2:** Patients' numerical velocity magnitude in the ascending aorta.

Diastolic pressure				Systolic pressure			
Inlet velocity (mm/s)		Mean velocity (mm/s)		Inlet velocity (mm/s)		Mean velocity (mm/s)	
Nondilated	Dilated	Nondilated	Dilated	Nondilated	Dilated	Nondilated	Dilated
119.69	135.88	29.34	23.14	166.67	185.10	40.50	31.57
136.27	137.17	29.48	34.97	194.24	230.54	42.39	53.81
144.56	169.83	31.56	40.87	197.95	230.85	44.91	57.91
146.73	174.11	34.12	41.00	210.61	231.60	47.41	58.37
150.44	175.34	34.48	42.60	210.86	241.72	49.21	59.78
153.99	179.42	35.36	46.67	214.02	242.13	49.77	61.86
160.59	187.01	46.60	51.58	219.28	249.11	63.01	68.40
160.94	189.70	46.64	53.70	238.50	269.57	68.07	72.33
184.46	190.39	50.92	57.95	247.49	302.21	68.77	90.59
207.19	262.59	60.00	62.17	271.10	391.20	78.55	90.71
Mean ± SD							
156.49 ± 24.57	180.14 ± 34.98	39.85 ± 10.48	45.47 ± 11.54	217.07 ± 29.49	257.40 ± 55.70	55.26 ± 13.19	64.53 ± 17.49
Shapiro-Wilk test							
0.54	0.04	0.12	0.92	0.93	0.03	0.18	0.39
Levene's test							
0.64		0.79		0.44		0.71	
*p* value							
0.097		0.270		0.058		0.197	

Inlet velocity means the average velocity over inlet surface. Mean velocity means the average velocity of all nodes in the ascending aorta. Value ordered from min to max in each group. *p* value stands for Students' *t*-test between the nondilated and dilated groups.

**Table 3:** Patients' numerical displacement results in the ascending aorta.

Systolic pressure			
Max displacement (mm)		Mean displacement (mm)	
Nondilated	Dilated	Nondilated	Dilated
0.107	0.113	0.049	0.042
0.113	0.205	0.054	0.088
0.126	0.220	0.057	0.101
0.167	0.231	0.076	0.108
0.189	0.243	0.089	0.111
0.194	0.250	0.091	0.115
0.197	0.397	0.092	0.193
0.249	0.445	0.117	0.208
0.257	0.454	0.127	0.221
0.372	0.514	0.175	0.236
Mean ± SD			
0.197 ± 0.081	0.307 ± 0.133	0.093 ± 0.039	0.142 ± 0.066
Shapiro-Wilk test			
0.27	0.25	0.31	0.22
Levene's test			
0.19		0.17	
*p* value			
0.038		0.056	

Value ordered from min to max in each group. *p* value stands for Student's *t*-test between the nondilated and dilated groups.

**Table 4:** Patients' numerical stress results in the ascending aorta.

Diastolic pressure				Systolic pressure			
Max stress-P1 (kPa)		Mean stress-P1 (kPa)		Max stress-P1 (kPa)		Mean stress-P1 (kPa)	
Nondilated	Dilated	Nondilated	Dilated	Nondilated	Dilated	Nondilated	Dilated
158.43	172.41	15.63	20.86	250.23	272.17	28.82	34.09
166.76	265.08	19.77	28.36	272.75	440.02	31.81	61.82
338.61	302.73	21.37	39.39	581.86	634.24	39.85	76.13
376.63	337.26	25.15	48.35	608.32	687.91	40.43	76.98
436.37	413.98	32.47	49.40	636.05	702.11	60.17	79.49
438.42	441.59	35.04	52.45	753.76	711.01	66.93	89.61
464.80	464.23	41.41	56.66	771.48	764.58	71.67	121.22
546.69	594.70	41.77	71.14	803.06	846.03	82.54	134.07
578.26	617.56	55.33	73.59	959.85	884.96	89.44	140.91
649.82	793.40	79.77	90.10	963.93	1112.47	123.10	165.40
Mean ± SD							
415.48 ± 162.40	440.29 ± 186.55	36.77 ± 19.35	53.03 ± 21.09	660.13 ± 247.47	705.55 ± 232.08	63.47 ± 29.77	97.97 ± 40.76
Shapiro-Wilk test							
0.57	0.90	0.20	0.95	0.27	0.83	0.49	0.75
Levene's test							
0.65		0.75		0.65		0.38	
*p* value							
0.755		0.089		0.677		0.044	

Value ordered from min to max in each group. *p* value stands for Student's *t*-test between the nondilated and dilated groups.

## Data Availability

The datasets generated during and/or analyzed during the current study are available from the corresponding author on reasonable request.
